# Contrast-enhanced mammography-guided biopsy: technical feasibility and first outcomes

**DOI:** 10.1007/s00330-022-09021-w

**Published:** 2022-07-27

**Authors:** R. Alcantara, M. Posso, M. Pitarch, N. Arenas, B. Ejarque, V. Iotti, G. Besutti

**Affiliations:** 1 Radiology and Nuclear Medicine Department, Hospital del Mar, Parc de Salut Mar, Passeig Marítim de la Barceloneta, 25-29, 08003 Barcelona, Spain; 2 Department of Medicine, Universitat Autònoma de Barcelona, Barcelona, Spain; 3 Department of Epidemiology and Evaluation, IMIM (Hospital del Mar Medical Research Institute), Passeig Marítim de la Barceloneta, 25-29, 08003 Barcelona, Spain; 4 Radiology Unit, Department of Diagnostic Imaging and Laboratory Medicine, Azienda USL-IRCCS di Reggio Emilia, Viale Risorgimento 80, 42123 Reggio Emilia, Italy; 5 Department of Medical and Surgical Sciences, University of Modena and Reggio Emilia, Modena, Italy

**Keywords:** Breast neoplasms, Mammography, Contrast media, Biopsy, Feasibility studies

## Abstract

**Objectives:**

To evaluate the feasibility of contrast-enhanced mammography (CEM)-guided biopsy at Hospital del Mar, a Spanish university hospital.

**Methods:**

We retrospectively reviewed all consecutive women with a suspicious enhancing finding eligible for CEM-guided biopsy, who were prospectively enrolled in a pre-marketing clinical validation and feasibility study (October 2019 to September 2021). CEM-guided biopsy is a stereotactic-based procedure that, by using intravenous iodinated contrast media administration and dual-energy acquisition, provides localisation of enhancing lesions. All the biopsies were performed using a vacuum-assisted device. We collected procedural characteristics (patient position and type of approach), and histopathological results. Feasibility endpoints included success (visualisation of the enhancing lesion, post-procedural biopsy changes and clip placement), procedural time, number of scout acquisitions and complications.

**Results:**

A total of 66 suspicious enhancing lesions (18.0% foci, 44.0% mass, 38.0% non-mass enhancement; median size 8.5 mm) in 64 patients (median age 59 years, mostly minimal [48.4%] or mild [32.8%] background parenchymal enhancement) were referred for CEM-guided biopsy in the study period. The success rate was 63/66 (95.4%). Amongst successful procedures, patients were most frequently seated (52/63, 82.5%) and the preferred approach was horizontal (48/63, 76.2%). Median total time per procedure was 15 min. Median number of acquisitions needed before targeting was 2 (range 1–4). Complications consisted of hematoma (17/63, 27%) and vasovagal reaction (2/63, 3.2%). At histology, the malignancy rate was 25/63 (39.7%).

**Conclusion:**

In this first patient series, CEM-guided breast biopsy was feasible, with success and complication rates similar to those previously reported for magnetic resonance guidance.

**Key points:**

• *CEM may be used to guide biopsy of enhancing lesions through a stereotactic-based procedure combined with intravenous iodinated contrast media administration and dual-energy acquisition.*

• *In this first patient series (n = 64), the success rate of CEM-guided biopsy was above 95%, the only complications were hematoma (22.2%) and vasovagal reaction (3.2%), and median total time per procedure was 15 min.*

• *CEM-guided biopsy is feasible and could potentially be a widely available biopsy technique for enhancing-only lesions.*

**Supplementary Information:**

The online version contains supplementary material available at 10.1007/s00330-022-09021-w.

## Introduction

Breast biopsy can be guided by different imaging techniques. The preferred first-line modality is ultrasound (US) but when suspicious lesions, such as architectural distortions or calcifications, are not visible on US, stereotactic or digital breast tomosynthesis (DBT) guidance is used.

Some breast lesions can be depicted only by imaging modalities able to detect tumour neoangiogenesis, visualised as suspicious contrast enhancement after the injection of intravenous contrast media [1]. For these ‘*enhancing-only*’ lesions, magnetic resonance imaging (MRI) guidance has, to date, been the only option in percutaneous biopsy, if there is no correlating finding in conventional second-look examinations [2–5]. However, MRI-guided biopsy is not widely available, is expensive and is not feasible in patients with contraindications such as claustrophobia, anthropometric limitations or metal devices.

Contrast-enhanced mammography (CEM) is an emerging imaging tool that, similar to MRI, allows visualisation of breast tumour neovascularisation [6, 7]. The technique consists of the acquisition of a dual-energy mammogram 2 min after intravenous injection of iodinated contrast media [8]. CEM displays both a low-energy image, similar to regular digital mammography, providing morphologic information, and a recombined image, depicting contrast media distribution, including local changes in breast perfusion presumably caused by tumour angiogenesis. The most common indications for CEM are inconclusive findings [9, 10], pre-operative staging [11, 12] and response monitoring [13]. Another potential indication is as a screening tool for women with increased risk of breast cancer [14–16]. Compared with MRI, CEM is less expensive [17] and seems to be preferred by women as it is faster and offers more patient comfort [18, 19].

There is scarce information in the literature on enhancing-only lesions detected by CEM. Amongst 839 women who underwent CEM after a screening recall, 70 enhancing-only lesions were found in 65 (7.7%) women. Of these 70 lesions, 38 (54.3%) subsequently proved to be malignant [10].

CEM guidance is possible due to a specific biopsy hardware add-on to the mammographic equipment, in addition to a dedicated software upgrade that applies the dual-energy acquisition to the stereotactic technique; currently, only one device is commercially available.

Due to the above-mentioned similarities between the 2 techniques, it is reasonable to assume that CEM-guided biopsy may be a valid alternative to MRI guidance. The purpose of this study was to evaluate the feasibility of CEM-guided biopsy in terms of its success rate (visualisation of the suspicious lesion during the procedure, allowing adequate targeting and sampling), procedure time and complication rate amongst the first described series of patients undergoing this procedure.

## Methods

### Study design and population

This was a retrospective review of a cohort prospectively enrolled in a pre-marketing clinical validation and feasibility study performed at Hospital del Mar, a university hospital in Barcelona, Spain (ClinicalTrials.gov ID: NCT05250674). We included all consecutive adult women presenting with a clinical indication for CEM-guided breast biopsy and considered eligible for this procedure as per standard of care between October 2019 and September 2021. CEM-guided biopsy was clinically indicated for suspicious enhancing lesions, not reliably identified by other morphological breast imaging techniques, including ‘second-look’ US or FFDM. Exclusion criteria were contraindications to iodinated contrast media. The study was approved by the hospital ethics committee (protocol number 2019/8890) and all patients provided written informed consent. A pre-marketing version of the Pristina Serena Bright™ GE Healthcare system had been under clinical validation in this study since 2019. The system received CE mark and Food and Drug Administration (510k) pre-marketing notification [20]. All the biopsy procedures were carried out using a 7- or 10-gauge (G) vacuum-assisted biopsy (VAB) needle.

### CEM-guided biopsy

**Fig. 1:**
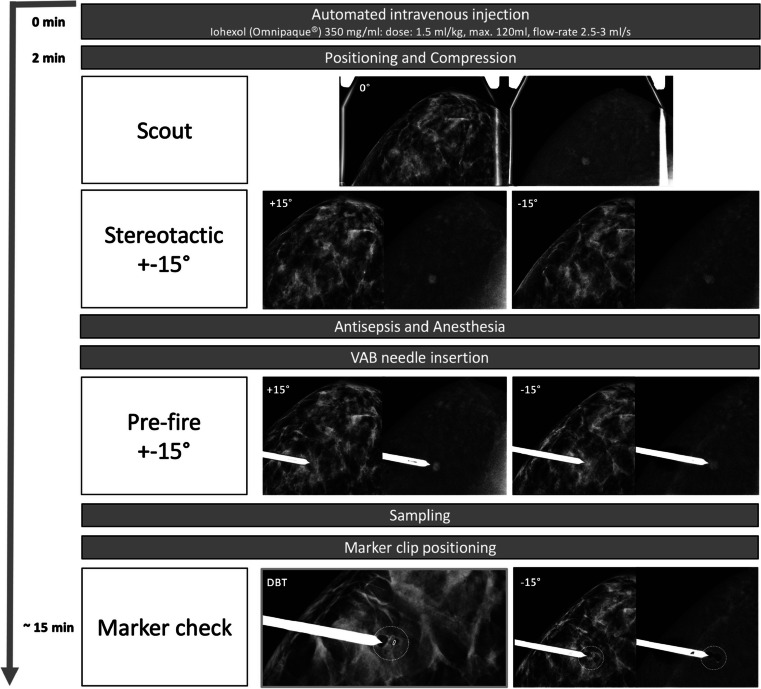
Flow diagram representing CEM-guided breast biopsy procedural steps. Three-dimensional localisation of enhancing lesions was provided by information obtained from stereotactic pairs of two-dimensional dual-energy scout and angulated images acquired under the same breast compression

The procedure workflow is depicted in Fig. 1.

The first step of any mammographic-guided procedure should be a prior complete review of the case to define the optimal approach, based on the location of the lesion in the breast and the patient’s characteristics, such as breast thickness and any potential physical limitations. As the evaluated equipment is an upright biopsy add-on unit, the patient can be seated or lying in a lateral decubitus position, with the help of a decubitus breast imaging table. The same dedicated needle support can be used for both the vertical and horizontal approach. The optimal approach should be planned on the diagnostic CEM that leads to the indication for CEM-guided biopsy. When the suspicious enhancing finding was originally seen on MRI, a pre-procedural CEM was performed to allow correct planning.

CEM-guided biopsy uses the principle of conventional stereotactic guidance, with the additional preliminary step of intravenous iodinated contrast media administration. As with diagnostic CEM, a 2-min interval between contrast administration and compression is necessary to avoid interference with blood inflow to the breast, allowing adequate lesion enhancement. The focal compression applied during the biopsy procedure seems to minimise lesion washout and contrast uptake is visible for up to 10 min, which is sufficient for proper targeting.

At the end of the required 2-min interval, the patient is positioned, the area where the target lesion is expected to be found is centred on the compression paddle, and a dual-energy image is acquired at 0°. Once the inclusion of the enhancement in the scout image has been confirmed, a pair of dual-energy angled views is acquired at ± 15°. In each of the angled images, the target is identified and marked, so that the device can calculate the 3 location coordinates (*X*, *Y* and *Z*), allowing the biopsy needle holder to be automatically positioned.

After antisepsis and local anaesthesia, the needle is inserted into the breast, until reaching the limit point defined by the support itself, when the tip of the needle would face the target. A pre-fire stereo pair of dual-energy views is then performed to confirm that the target is in front of the needle tip; otherwise, minimal coordinate adjustments may improve precision. Next, once the fire-forward is activated, the sampling is carried out with the available vacuum system device. After sampling, the biopsy bed will be contaminated by blood with contrast media, so the pre-fire image will be the last to benefit from the dual technique.

It is highly important to place a radiological marker on the biopsy bed in order to evaluate procedural effectiveness and enable further localisation if the results reveal malignancy. It is recommended to check marker release with a DBT scout image, since this allows its precise location with a single acquisition, and therefore with a lower average glandular dose (AGD) than stereotactic acquisitions [21]. At the end of the procedure, the correct location of the radiological marker is confirmed, with the usual 2 mammographic views (mediolateral and craniocaudal), which are compared with the pre-procedural low-energy images.

### Data collection

Clinical data of the included patients and suspicious lesions were collected, including patient age, breast density, background parenchymal enhancement (BPE) and the size and type of enhancement of the target lesion. Breast density was visually assessed on the low-energy image of the diagnostic CEM according to breast composition categories of the American College of Radiology (ACR) BI-RADS® mammography lexicon [22] and collected from the radiology report. BPE was visually graded in the diagnostic recombined image into 4 categories (minimal, mild, moderate and marked), according to the BI-RADS® MRI lexicon [23], applied for CEM [24]. Similarly, the reported size of the suspicious lesion was collected and the type of enhancement was classified in focus or foci (< 5 mm), mass enhancement (> 5 mm) and non-mass enhancement [23, 24].

Procedural characteristics, i.e. patient position (seated/lying), the kind of approach (horizontal/vertical) and needle gauge, were collected for each procedure.

For successful procedures, we collected the histopathological results on the biopsy specimen, as well as the kind of treatment undergone by patients. For patients who underwent surgery, we collected the pathological results of the surgical specimen.

### Endpoints and data analysis

To evaluate the feasibility of CEM-guided biopsy, various endpoints were taken into account.

The success rate was calculated. *Procedural success* was defined as the visualisation of the suspicious enhancing lesion during procedural CEM, allowing adequate targeting and sampling, together with post-procedure imaging showing biopsy changes at the site of the intended lesion and with the clip in place or nearby in sequential mammography. Other cases were classified as *cancelled*, when the enhancing lesion visible at diagnostic CEM was not visible on procedural CEM, leading to non-performance of biopsy and short-term interval follow-up. Biopsies were classified as *unsuccessful* when the enhancing lesion was less visible on all procedural CEM views, limiting proper target selection, but the biopsy could still be performed by using another reference and guidance.

Procedural time was registered, considering the time span from contrast administration to clip placement scout view, immediately prior to breast decompression. Moreover, the number of scout acquisitions needed to represent the enhancing target in the field of view was collected. The complication rate was calculated, including immediately visible hematomas, vasovagal reactions, allergic reactions and late complications such as hematomas or infection.

For patients who underwent surgery or percutaneous VAE, the upgrade rate was calculated as the percentage of cases originally graded as B3 on the biopsy specimen, according to the NHS Breast Screening Programme pathology classification [25, 26], and then found to be malignant on the surgical or excision specimen, as well as cases diagnosed as ductal carcinoma in situ (DCIS) on biopsy and upstaged to invasive ductal carcinoma (IDC) on the surgical specimen.

## Results

### Study population

**Fig. 2:**
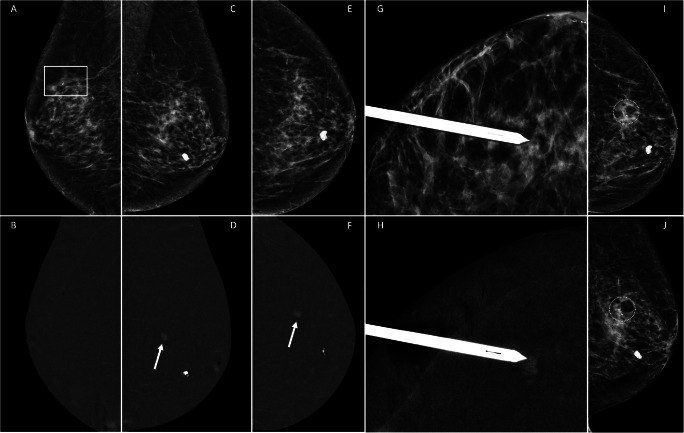
An asymptomatic 77-year-old patient with a history of right breast-conserving surgery was referred for complementary evaluation after detection of a suspicious scar in a routine external centre ultrasound (images not available). Low-energy CEM MLO view (**A**) of the right breast shows a stable architectural distortion related to the postsurgical scar (rectangle) with no enhancement on the recombined image (**B**). Left breast recombined images (**D** and **F**) show an incidental 7-mm enhancing mass (arrow) not seen on low-energy images (**C** and **E**, respectively) or ultrasound. Left breast CEM-guided biopsy was performed (**G** and **H**). The correct location of the radiological marker was confirmed, with the usual 2 mammographic craniocaudal and mediolateral views (**I** and **J**). Pathology report: intraductal papilloma. The lesion was further assessed with a DBT-guided vacuum-assisted excision (VAE)

**Fig. 3:**
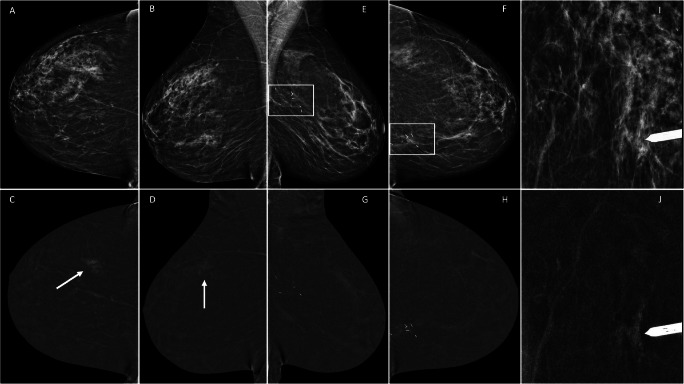
A 53-year-old patient with a history of left breast–conserving surgery was referred for complementary evaluation after questionable changes in clinical examination of the left breast. Diagnostic CEM was performed, together with ultrasound. Low-energy CEM mediolateral oblique (**E**) and craniocaudal (**F**) views of the left breast show a stable postsurgical scar (rectangle) with no enhancement at recombined images (**G** and **H**, respectively). Right breast recombined images (**C** and **D**) show an incidental 26-mm non-mass enhancing finding (arrow) not clearly seen on low-energy images (**A** and **B**, respectively), DBT or ultrasound (not shown). Right breast CEM-guided biopsy was performed (**I** and **J**). Pathology report: High-grade triple negative ductal carcinoma in situ (DCIS). The lesion was further assessed with wire needle-guided surgical conservative treatment

From October 2019 to September 2021, 64 patients were referred for CEM-guided breast biopsy. Of these, 2 underwent 2 biopsies for 2 different suspicious lesions (performed on the same day, in one patient with only one contrast administration), making a total of 66 procedures (Table 1). The median age of the included patients was 59 years, and breast density was predominantly classified as B (35.9%) and C (59.4%), whilst BPE was mostly minimal (48.4%) or mild (32.8%). Suspicious enhancing lesions were mass enhancement in 43.9% of the procedures, focus/foci in 18.2% and non-mass enhancement in 37.9% (Figs. 2 and 3). The median size of the suspicious lesion was 8.5 mm. Median time from diagnostic CEM to CEM-guided biopsy was 12 (6–21) days. For the 10 patients with an MRI-detected suspicious lesion, the CEM-guided biopsy was performed 15 (10–23) days after the MRI.

**Table 1:** Clinical characteristics of the included patients and respective suspicious lesions for which CEM-guided biopsy was suggested. *According to ACR BI-RADS® breast composition categories. **Applied from ACR BI-RADS® MRI lexicon. *IQR*, interquartile range

		Patients undergoing CEM-guided biopsy (*n* = 64)
Age, years, median (IQR, range)		59 (IQR 15; range 22–88)
Breast density*, *n* (%)	A	0
	B	23 (35.9%)
	C	38 (59.4%)
	D	3 (4.7%)
CEM background parenchymal enhancement, *n* (%)	Minimal	31 (48.4%)
	Mild	21 (32.8%)
	Moderate	9 (14.1%)
	Marked	3 (4.7%)
		CEM-guided biopsies (*n* = 66)
Clinical indication for diagnostic CEM	Problem-solving technique	17 (25.8%)
	Screening recall	18 (27.3%)
	Staging (after MRI)	11 (16.7%)
	Symptomatic patients	20 (30.3%)
Suspicious finding for biopsy**, *n* (%)	Mass enhancement	29 (43.9%)
	Focus/Foci	12 (18.2%)
	Non-mass enhancement	25 (37.9%)
Size of suspicious finding, mm, median (IQR, range)		8.5 mm (IQR 14, range 3–60)

*IQR* Interquartile range

### Procedural characteristics and feasibility

**Fig. 4:**
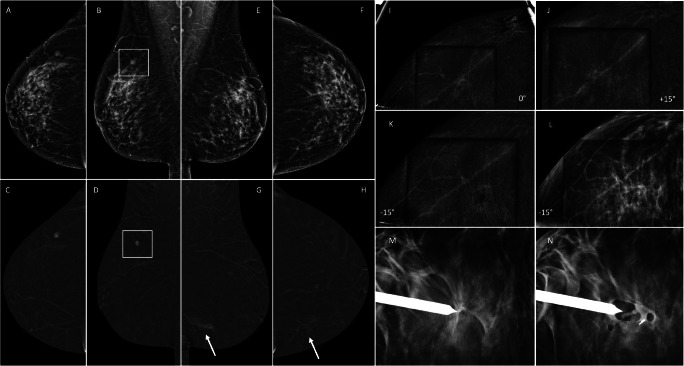
Unsuccessful CEM guidance due to decreased enhancement. A 50-year-old asymptomatic patient was referred from the breast cancer screening programme due to a mass in the upper outer quadrant of the right breast (square). A CEM was performed (**A**–**H**), and an intense mass enhancement (square in **B** and **D**) led to an ultrasound-guided biopsy of this lesion, which was diagnosed as a complex fibroadenoma. As an incidental finding, there was a mild 15-mm non-mass enhancement in the inferior inner quadrant of the left breast (arrow in **G** and **H**), with no clear correlation in low-energy images (**E** and **F**). The patient was scheduled for a CEM-guided procedure. In the scout at 0° (**I**), there was mild enhancement but the finding was less visible in recombined angled views (**J** and **K**). A DBT scout confirmed a small distortion in the location of the enhancement, allowing biopsy (**M** and **N**). Pathology reported a grade I invasive ductal carcinoma

Of the 66 suspicious lesions with an indication for CEM-guided biopsy, the procedure was successful in 63 (95.4%) lesions (Table 2). For the remaining 3 lesions, the enhancement classified as suspicious at diagnostic CEM was not clearly visible at procedural CEM. In 2 of these 3 patients, the biopsy was cancelled. In the remaining patient, who had a mild BPE, although the enhancement was not clearly visible in all views, a small distortion was seen at the time of the procedure with a complementary DBT scout, and biopsy was performed under DBT guidance, resulting in IDC (Fig. 4). Of the 2 patients with cancelled biopsies, one had a further follow-up CEM after 6 months showing no suspicious findings, whilst the second had no available follow-up CEM, although there was no evident suspicious enhancement at the CEM retest performed on the same day as the planned procedure (Supplementary Fig. 1). The original suspicious findings in these 2 patients were a 55-mm non-mass enhancement and a 6-mm mass enhancement, and BPE was moderate in the first and mild in the second.

**Table 2:** Procedural success rate and characteristics of successful procedures. *Time per procedure was available in 59 procedures; data on time were not collected in the remaining 4 procedures nor could they be retrospectively obtained

		CEM-guided biopsies (*n* = 66)
Success rate, *n* (%)	Successful	63 (95.4%)
	Unsuccessful	1 (1.5%)
	Cancelled	2 (3.0%)
		Successful CEM-guided biopsies (*n* = 63)
Total time per procedure, min, median (IQR, range)		15 min (IQR 4, range 9–25) (*n* = 59, 4 missing) *
Number of scout acquisitions per procedure, median (IQR, range)		2 scouts (IQR 1, range 1–4) (*n* = 62, 1 missing)
Position on the chair, *n* (%)	Seated	52 (82.5%)
	Lying	11 (17.5%)
Approach, *n* (%)	Horizontal	48 (76.2%)
	Vertical	15 (23.8%)
Needle gauge, *n* (%)	10G	52 (82.5%)
	7G	11 (17.5%)
Biopsy complications, *n* (%)	No complications	44 (69.8%)
	Vasovagal reactions	2 (3.2%)
	Early hematomas	14 (22.2%)
	Late hematomas	3 (4.8%)

*IQR* Interquartile range

As for the procedural characteristics of successful CEM-guided biopsies, the patients were most frequently seated (*n* = 52, 82.5%) and the preferred approach was horizontal (*n* = 48, 76.2%). The median total time per procedure was 15 min. The number of acquisitions needed before the targeting was between 1 and 4 (median 2 acquisitions). In most procedures (*n* = 4, 69.8%), no complications were registered. Self-limiting early hematomas were recorded in 14 (22.2%) patients and vasovagal reactions in 2 (3.2%). Late complications consisted of only moderate hematomas (3/63, 4.8%). No severe complications or allergic reactions were registered.

### Pathology results of successful procedures

**Fig. 5:**
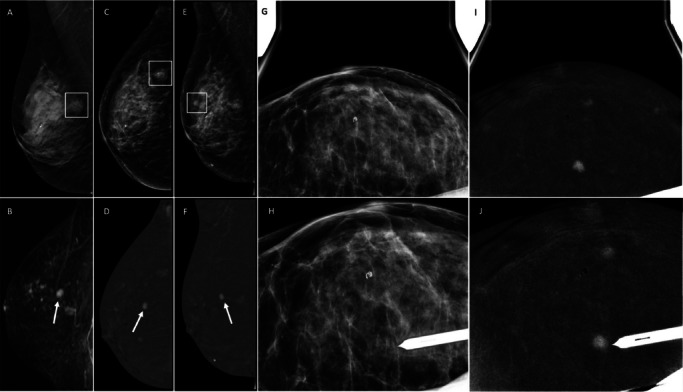
A 47-year-old patient with a history of right breast–conserving surgery 11 years ago and bilateral fibroadenomas (squares in **A**, **C** and **E**). Routine MRI follow-up (sagittal 3D Maximum Intensity Projection subtraction, **B**) showed a new right breast mass (arrow) not seen on second-look ultrasound or DBT (mediolateral oblique planes, **A**). Right breast recombined images (**D** and **F**) show the 8-mm enhancing mass (arrow), not seen on low-energy images (**C** and **E**). Right breast CEM-guided biopsy was performed (**G**–**J**). Pathology report: invasive lobular carcinoma. The patient underwent a mastectomy

Of the 63 lesions that underwent successful CEM-guided biopsy, 2 (3.2%) were B1 on biopsy specimen, 26 (41.3%) were B2 (predominantly fibroadenomas, fat necrosis and adenosis/sclerosis), 10 (15.9%) were B3 (predominantly atypical ductal hyperplasia) and 25 (39.7%) were B5, including IDC (*n* = 10), DCIS (*n* = 7), a combination of IDC and DCIS (*n* = 1) and invasive lobular carcinoma (*n* = 7) (Fig. 5). The median size of the suspicious enhancement according to histopathological diagnosis is reported in Table 3. After radiological-pathological correlation, only 2 patients with discordant results were referred to short-term follow-up; one of these showed no suspicious enhancement after 6 months and the other showed remaining enhancement at 6 months, resulting in a re-biopsy close to the clip, still with B2 histopathological results. Of the 35 lesions that were treated, whether surgically or through percutaneous VAE, 6 (17.1%) were upgraded, including 4/10 B3 lesions which were upgraded to malignancy, and 2/7 DCIS which were upstaged to IDC (Table 3).

**Table 3:** Pathology results on biopsy specimen for successful biopsies, type of treatment and pathological analysis of surgical specimen for surgically treated lesions

			Successful CEM-guided biopsies (*n* = 63)
Pathology on biopsy specimen, *n* (%)	B1	Normal tissue	2 (3.2%)
	B2		25 (39.7%)
		Adenosis/sclerosis	5
		Fibroadenoma	7
		Usual ductal hyperplasia	3
		Fat necrosis	7
		Periductitis	3
	B3		11 (17.4%)
		Classical lobular neoplasia	3
		Atypical ductal hyperplasia	6
		Papilloma	1
		Phyllodes	1
	B4		0
	B5		25 (39.7%)
		IDC	10
		DCIS	7
		IDC + DCIS	1
		ILC	7
	Size of the suspicious lesion by pathology; median (IQR) (mm)	B1 (*n* = 2)	7.5 (6–9)
		B2 (*n* = 25)	8 (6–14)
		B3 (*n* = 11)	10 (8–18)
		B5 (*n* = 25)	9 (6–23)
Patient after biopsy, *n* (%)	Surgery	Conservative	25 (39.7%)
		Mastectomy	9 (14.3%)
	DBT-guided VAE Follow-up		1 (1.6%)
		Short-term follow-up	3 (4.8%)
		Routine follow-up	25 (39.7%)

*VAE* vacuum-assisted excision, *IQR* interquartile range, *DBT* digital breast tomosynthesis, *DCIS* ductal carcinoma in situ, *IDC* invasive ductal carcinoma, *ILC* invasive lobular carcinoma

## Discussion

This is the first report of a series of patients who received CEM-guided breast biopsy, a new approach that allows targeting of enhancing lesions and which may be a potential alternative to MRI guidance. In this series of 64 patients (with a total of 66 suspicious lesions), the success rate was above 95%. The preferred patient position was seated, and the preferred approach was horizontal. The median total time per procedure was 15 min, and the median number of acquisitions before the targeting was 2. The only complications were early or late hematoma (27%) and vasovagal reaction (3.2%).

MRI-guided biopsy is a safe and accurate technique but requires experience and the localisation process can be laborious and time-consuming, with a reported median of 35–41 min for imaging time and room occupancy of around 60–70 min [27–31]. Other major drawbacks are its limited availability and high costs [30].

The MRI-guided VAB success rate reported in the literature ranges from 87 to 98%, with some degree of procedure deferral due to absent enhancement or decreased visualisation [32–36]. The CEM-guided biopsy success rate in our case series was similar (95.4%). Of the 3 findings with absent or decreased visualisation at the time of the procedure, one was an IDC, whilst the follow-up available in one of the 2 patients with cancelled biopsies showed no suspicious lesions. Non-visualisation of a previously detected enhancing finding has been described in approximately 8–13% of MRI-guided biopsies [37–39], and possible explanations for these events, both for MRI and for CEM, are noncyclical hormonal factors, qualifying as BPE or focal inflammatory or fibrocystic changes that may be cleared out or scattered by the time of the procedure [39]. Despite the low cancer detection rate amongst the lesions leading to deferred MRI-guided biopsy (0–10%) [40], ACR Practice Parameters recommend a ‘short-term’ follow-up [4], and this practice was also applied to the 2 patients with cancelled biopsies.

In our case series, the malignancy rate at biopsy was almost 40%, falling within the range of the cancer detection rate reported in the literature for MRI-guided breast biopsy, ranging from 18 to 61% [33, 36, 41]. The rate of upgrade and upstaging to malignant disease after histological analysis of open surgical excision samples for the 35 treated patients was 17.1%. The upgrade rate for B3 lesions was 40% (4 of 10 cases), in line or slightly superior to rates described in the literature (12–38%) [37, 41, 42]. Of note, rates of upgrade to malignancy may be higher for MRI-guided biopsy, and presumably for CEM-guided biopsy, than for stereotactic and US-guided biopsy [4, 36].

**Fig. 6:**
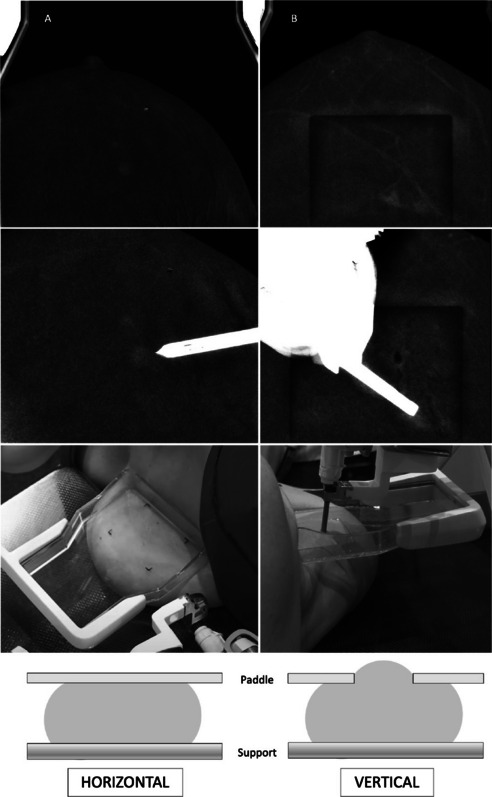
Vertical versus horizontal approach. **A** Horizontal approach (pathology report: invasive lobular carcinoma). **B** Vertical approach (pathology report: atypical ductal hyperplasia). The use of a plane compressor paddle with the horizontal approach provides better image quality and target visualisation

The horizontal approach was preferred in our series. The use of a plane compressor paddle with the horizontal approach offers better image quality and target visualisation. Conversely, the use of a fenestrated compressor paddle, necessary for the vertical approach, may result in artefacts in recombined images, due to differences in compressed breast thickness. Similarly, vertical access also has implications in the pre-fire view as the anaesthesia and VAB needle itself may interfere with the original target (Fig. 6).

The main complications were hematomas and vasovagal reactions, occurring in rates similar to those reported in the literature for other upright mammography-guided procedures [43–46], i.e. 1–5% for vasovagal reactions [43–45], and 2–83% for hematomas [45, 46], which are very common although not usually clinically relevant. No severe allergic reactions were observed, but this may be due to patient selection, since previous allergies to iodinated contrast media, also during diagnostic CEM, were an exclusion criterion. Irrespective of this consideration, the risk of contrast reaction is low with the type of contrast media used, less than 1%, and most events are mild and self-limiting [8, 10].

A limitation of the present study is the lack of information on AGD, since it can be argued that the main drawback of CEM guidance is radiation exposure. It is plausible that levels of exposure are acceptable, since previous data on diagnostic CEM have shown that the radiation dose remains below the threshold set by the European guidelines for screening mammography [8, 47]. In this study, the median number of scout views before targeting was low and we avoided additional dual-energy imaging after sampling. Nevertheless, this issue requires further investigation. In addition, the relatively small sample size of included women may limit the generalizability of our findings. However, we believe that the number of procedures assessed in this study was sufficiently large to provide valid information on the feasibility of CEM-guided biopsy. Further cohort studies with a larger number of cases should focus on evaluation of its effectiveness and on the potential negative influence on procedural success induced by masking of the target lesion due to BPE.

In conclusion, in this first series of patients, CEM-guided breast biopsy was a feasible technique that was easy to implement and perform and which showed good success and complication rates and short procedural time (15 min on average). Since CEM-guided breast biopsy has the potential to be an alternative to MRI-guided biopsy for enhancing-only lesions, further studies are needed to directly compare the 2 techniques in terms of patient preference and cost-effectiveness.

## Supplementary information

ESM 1 (DOCX 2356 kb)

## Abbreviations

AGD: Average glandular dose
BPE: Background parenchymal enhancement
CEM: Contrast-enhanced mammography
DBT: Digital breast tomosynthesis
DCIS: Ductal carcinoma in situ
FFDM: Full field digital mammography
IDC: Invasive ductal carcinoma
VAB: Vacuum-assisted biopsy
VAE: Vacuum-assisted excision

## Declarations

### Guarantor

The scientific guarantor of this publication is Dr. R. Alcantara.

### Statistics and biometry

One of the authors (M. Posso) has significant statistical expertise.

### Methodology

• Retrospective review of prospectively enrolled cohort

• Pre-marketing clinical validation

• Performed at one institution
