# Supplementary material for: Contrast-enhanced mammography-guided biopsy: technical feasibility and first outcomes
Source: Eur Radiol. 2022 Jul 27;33(1):417–28. doi: 10.1007/s00330-022-09021-w (PMC9755098; doi:10.1007/s00330-022-09021-w)
Supplement: Supplementary file 1 — (DOCX 2356 kb) [file 330_2022_9021_MOESM1_ESM.docx]

*
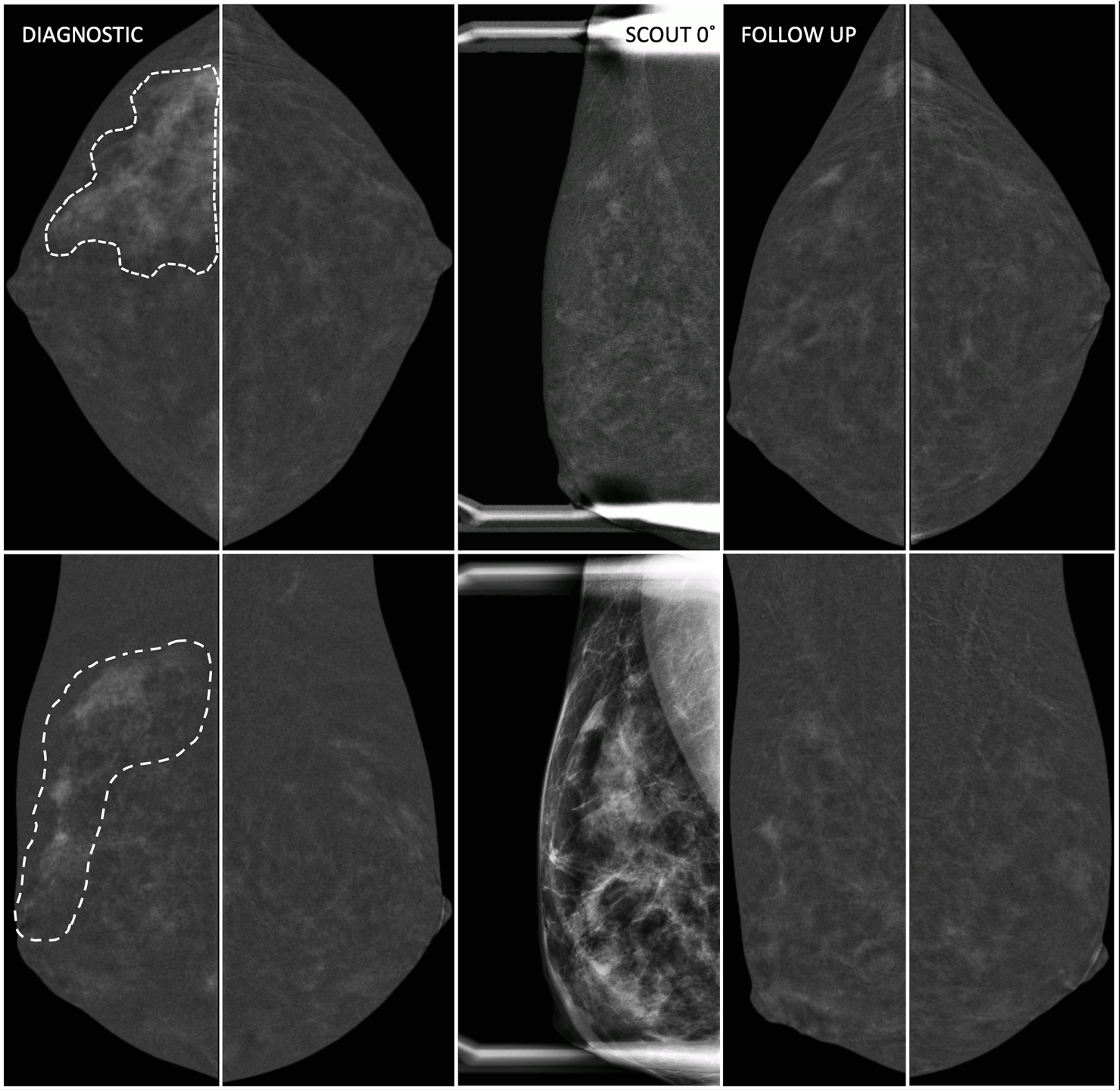
Supplementary Figure 1*: Withdrawn biopsy case due to lack of enhancement. A 40-year-old woman underwent workup of mastalgia and palpable lump in the outer quadrants of the right breast. On diagnostic CEM, an asymmetric 50-mm non-mass enhancement (dotted line) was seen, with no correlation in low-energy or ultrasound (not shown). CEM-guided biopsy was scheduled (2 weeks later). The initial finding was not reproducible on the day of the biopsy, or in a same day CEM retest and was therefore interpreted as asymmetric background parenchymal enhancement due to temporary focal inflammatory conditions. The procedure was withdrawn and a short-term follow-up was required.
